# Effects of ozone exposure on house dust mites

**DOI:** 10.1038/s41598-026-59397-8

**Published:** 2026-06-22

**Authors:** Isaac Seow, Zhen Yun Siew, Boon Xuan U, Chee-Mun Fang, Mun Seng Kan, Shew Fung Wong, Siew Tung Wong, Masita Arip, Husna Farhanah Ahmad, Suhaili Zainal Abidin, Kenny Voon

**Affiliations:** 1 Division of Biomedical Sciences, School of Pharmacy, University of Nottingham Malaysia, 43500 Semenyih, Malaysia; 2 Faculty of Information Science and Technology, Multimedia University, Bukit Beruang, 75450 Melaka, Malaysia; 3 School of Science, Monash University Malaysia, Bandar Sunway, Subang Jaya, 47500 Selangor Malaysia; 4 School of Medicine, IMU University, Kuala Lumpur, 57000 Malaysia; 5 Allergy and Immunology Research Centre, Institute for Medical Research, National Institutes of Health, Persiaran Setia Murni, Setia Alam, Shah Alam, 40170 Selangor Malaysia; 6 Acarology Unit, Infectious Diseases Research Centre, Institute for Medical Research, National Institutes of Health, Persiaran Setia Murni, Setia Alam, Shah Alam, 40170 Selangor Malaysia

**Keywords:** Allergy, House dust mites, Ozone, *Dermatophagoides farinae*, Next generation sequencing, Microbial diversity, Diseases, Environmental sciences, Medical research, Microbiology

## Abstract

House dust mites (HDMs), particularly *Dermatophagoides farinae*, are commonly found in household dust and play a key role in allergic diseases such as asthma and allergic rhinitis. Beyond clinical management, allergen removal strategies are crucial for improving quality of life. Hence, this study investigated the effects of ozone exposure on *D. farinae*, focusing on changes in protein expression, surface bacterial composition, mortality, and mobility. Mites were exposed to ozone concentrations of 0.05, 0.5, and 1 ppm for 24, 48, and 72 h in a controlled chamber, with non-exposed mites serving as controls. Western blotting using anti-Der f 1 and anti-Blo t 5 antibodies assessed changes in allergen profiles, while 16 S rRNA sequencing characterised changes in surface bacterial communities. Mortality was evaluated using 100 mites per group under varying exposure durations. To assess behavioural responses, a three-chamber mobility assay was conducted, where mites were placed in a central compartment flanked by no-ozone and low-ozone chambers, and their distribution was recorded after 72 h. Ozone exposure resulted in a concentration- and time-dependent reduction of Der f 1 protein intensity, suggesting allergen degradation. Surface bacterial profiling revealed distinct compositional shifts following ozone exposure. Mortality increased proportionally with ozone concentration and duration. In the mobility assay, mites predominantly remained in the no-ozone chamber, indicating avoidance of ozone. Collectively, these findings demonstrate that ozone exposure affects *D. farinae* at molecular, microbial, and behavioural levels, highlighting ozone’s potential role in modulating mite allergenicity and ecology.

**Supplementary Information:**

The online version contains supplementary material available at https://doi.org/10.1038/s41598-026-59397-8.

## Introduction

House dust mites (HDMs) such as *Dermatophagoides farinae (D. farinae)*, *Dermatophagoides pteronyssinus (D. pteronyssinus)*, *Euroglyphus maynei (E. maynei)*, and *Blomia tropicalis (B. tropicalis)* are widely acknowledged as culprits behind allergic diseases such as hay fever, rhinitis, asthma, and conjunctivitis^1^. They are present in almost every home and are mostly found in mattresses, carpets, sofas, and beddings because these places house the primary food source for HDMs, which are shed human scales^2^. Previous surveys in Malaysia demonstrated that HDMs are highly prevalent in households, with all mattress dust samples showing evidence of infestation^3^. This shows that HDMs are heavily populated in Malaysian households, and since their existence is greatly linked to a variety of allergic diseases, finding effective ways to inactivate or kill them are crucial for human health.

Control methods, including chemical acaricides, natural essential oils, frequent vacuuming, hot laundering, and sunlight exposure, can reduce mite levels, but many of them are labour-intensive, not cost-effective, with temporary effects and safety concerns^4^. Recently, ozone has emerged as a potential alternative due to its strong oxidising properties and ability to disrupt biological structures, making it a promising candidate for HDM control^5–7^. However, most ozone-based HDM studies have been conducted under controlled laboratory conditions, and their real-world applicability remains limited, especially considering that indoor ozone levels must comply with the Malaysian Ambient Air Quality Standard (MAAQS) limit of 0.1 ppm^8^. Most studies often overlook the persistence of allergenic proteins, such as Der f 1 and Der p 1, which remain in the environment even after mite elimination, as their allergens were primarily found in the faeces^9^. Therefore, it is crucial to investigate how ozone exposure affects the expression of these allergenic proteins. Notably, several studies have shown that ozone exposure can regulate protein expression, as demonstrated using Western blot analyses in different biological contexts^10–14^.

Besides, another gap is that the impact of ozone on surface bacteria within the mite micro-environment has largely been overlooked, despite the potential for microbial communities to influence allergen load and mite survival. Moreover, limited studies have explored behavioural responses such as ozone avoidance, which could provide a simple and non-chemical strategy to deter mites from human-occupied areas. To address these gaps, we evaluated the effects of ozone on *D. farinae* under controlled conditions. Western blotting was used to assess changes in major allergenic proteins, while mite mortality and behavioural preference assays evaluated ozone’s lethal and repellent effects. We also examined its impact on surface-associated bacteria to determine potential contributions to allergen reduction. These approaches provide a comprehensive evaluation of ozone as a multi-targeted strategy for HDMs control.

## Materials and methods

### Mites culture

**Fig. 1:**
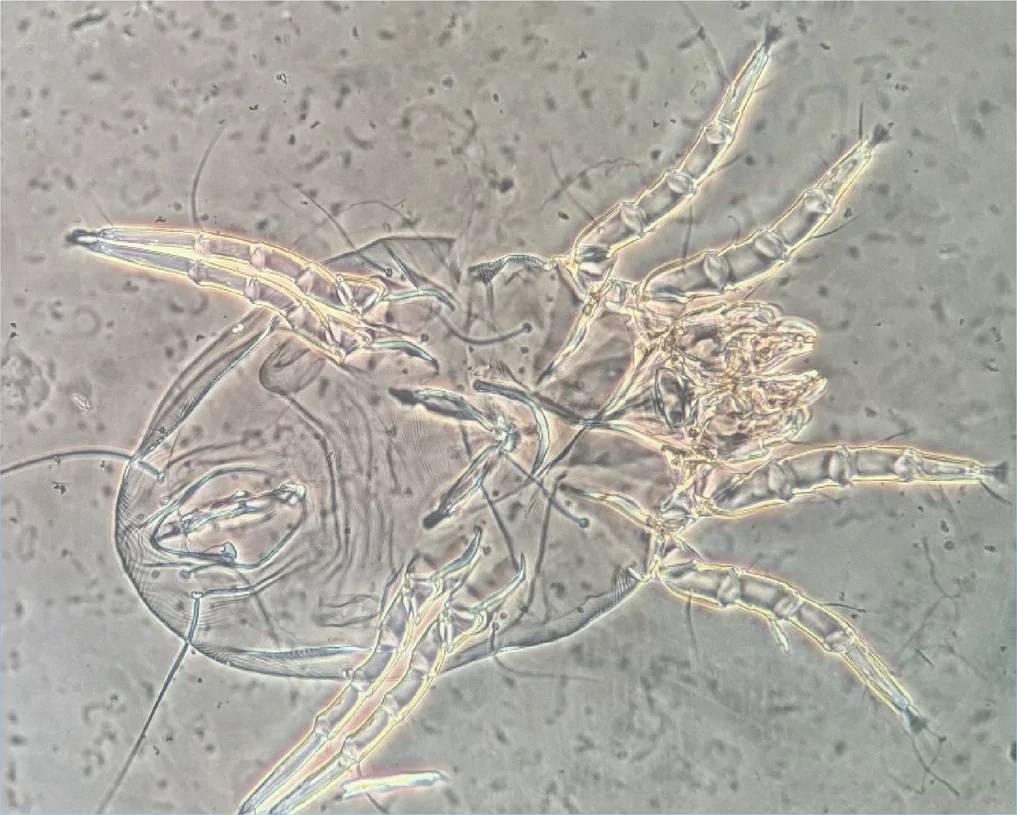
*D. farinae* female mite, at 40x/0.65 magnification, under a Nikon Eclipse phase contrast microscope.

*D. farinae* mites were obtained from colonies established since the 1960s in the Acarology Unit, Institute for Medical Research, Malaysia. The colonies were maintained in T75 culture flasks, fed with ground fish flakes (TetraMin Crisps, Germany), at an average temperature of 26 (± 2) °C and 70–75% relative humidity in a dark room. Routine maintenance of the mites by random sampling from the culture flasks to detect cross-contamination was performed by microscopic examination of the mites mounted in Hoyer’s medium^15^. Eight-legged, adult mites were used for all subsequent experiments. A microscopic image of the *D. farinae* mite is shown in Fig. 1.

### Ozone chambers construction

**Fig. 2:**
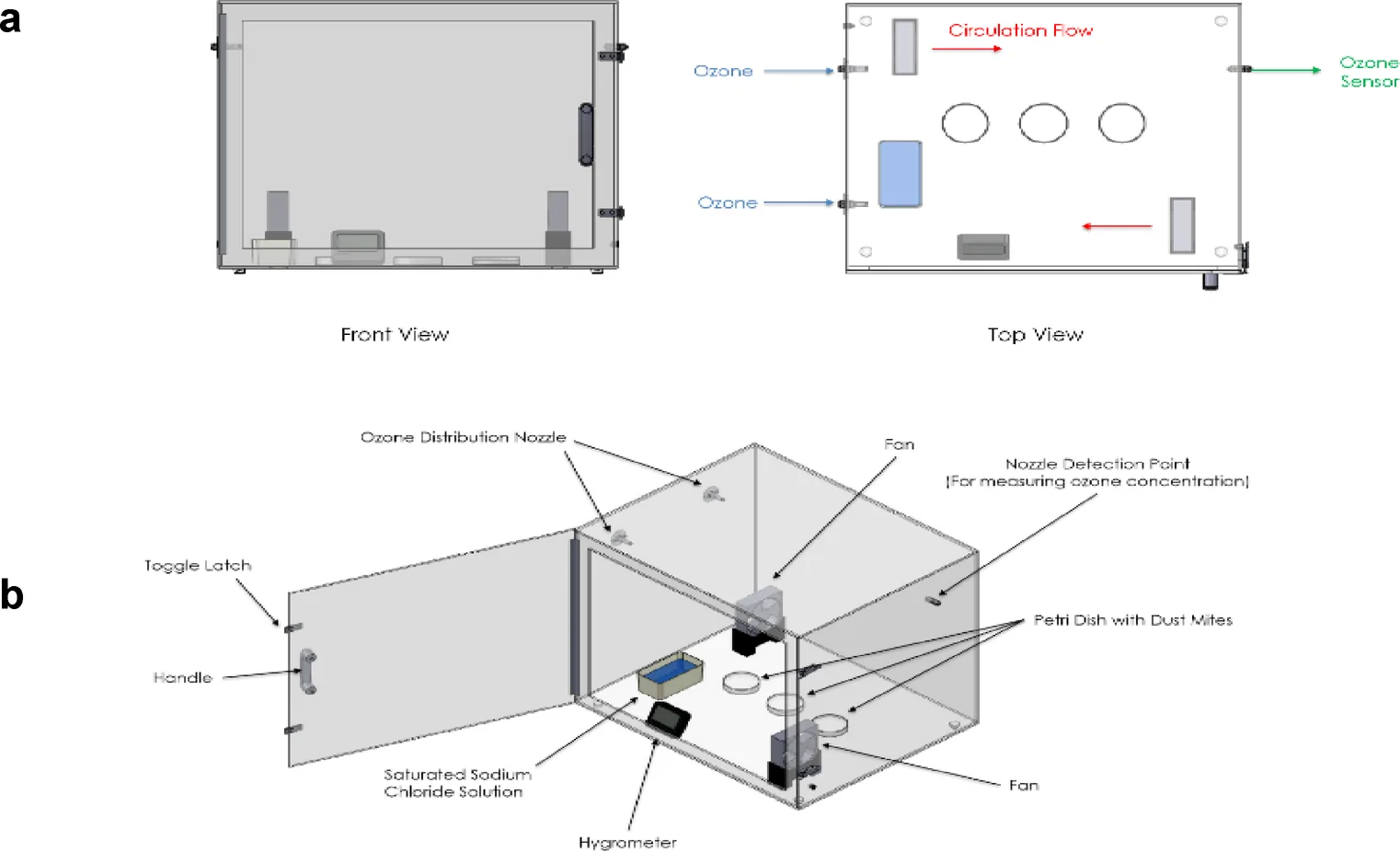
Design and airflow system of the mortality and protein-exposure chamber. (**a**) Isometric view of the custom-designed exposure chamber (AutoCAD schematic). (**b**) Labelled diagram showing internal layout and components of the chamber.

**Fig. 3:**
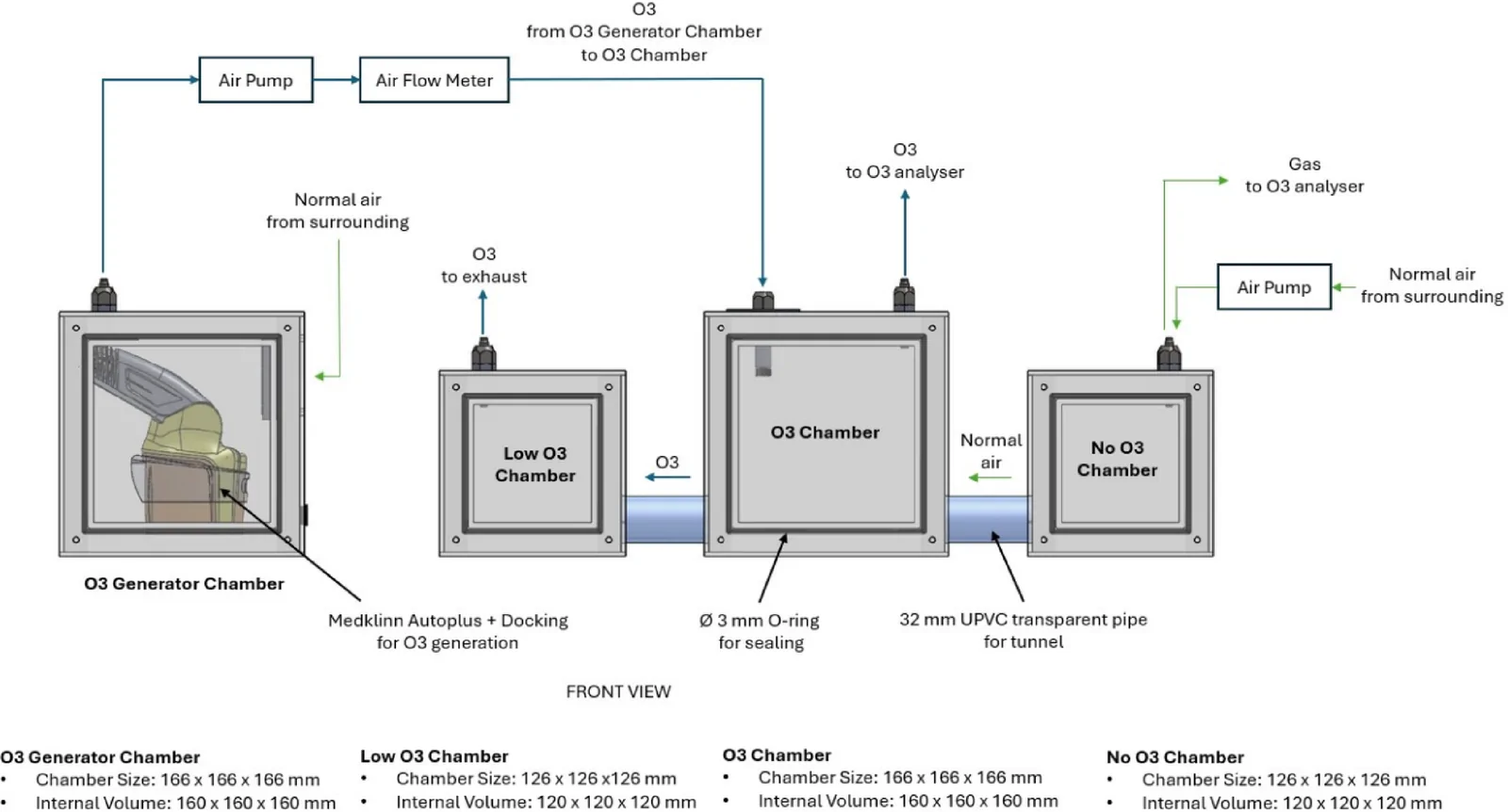
Design of the mobility assessment chamber system. Front view of the mobility assessment chambers illustrating the components, specifications, airflow pathway, and directional flow across the compartments.

Two customised airtight chambers were constructed based on a previous design with modifications (Abidin & Ming, 2012): one for assessing mite mobility under ozone exposure and another for evaluating mortality and collecting mites for protein analysis. The design of the chambers was drawn using AutoCAD (Autodesk Inc., USA) and is shown in Figs. 2 and 3.

For the mortality and protein-exposure chamber, a customised Medklinn ozone generation system was constructed with patented Cerafusion™ technology, which produces ozone at 0.05, 0.5, and 1.0 ppm, using a 1 g/hr generator with a controlled gas flow of 0.05–0.25 L/min. The mobility assay chamber employed a Medklinn Autoplus model (Medklinn International, Malaysia) with a 6 mg/hr ozone generation capacity to maintain concentrations of up to 1.0 ppm at a flow rate of 1 L/min. In both setups, ozone levels were continuously monitored with a Standard Enclosure Model 106-L Ozone Monitor™ (2B Technologies, USA) operating under the Federal Equivalent Method (FEM), using closed-loop feedback to regulate ozone generation^16^. The ozone generator was automatically switched on/off within a predefined deadband to maintain the target concentration. This resulted in minor oscillations within a narrow tolerance band, and ozone exposure was defined as the time-weighted mean concentration. Stability and control performance were verified via continuous logging using a HOBO data logger (Onset Computer Corporation, USA).

The mortality and protein-exposure chamber consisted of a single unit with two internal fans to ensure uniform ozone distribution. Relative humidity (75–80%) was maintained using a saturated sodium chloride solution, and temperature was controlled at 26 ± 2 °C, both monitored with a digital hygrometer. The mobility assay chamber was constructed with three interconnected acrylic compartments, allowing mites to move freely between sections with different ozone concentrations, while maintaining the same humidity and temperature conditions. Importantly, the tri-ozone chamber was designed to generate a controlled ozone gradient using balanced airflow while maintaining near-atmospheric pressure and directional flow. The ozone level gradient was verified using an additional ozone analyser: the no-ozone chamber showed concentrations below the detection limits, while the low-ozone chamber consistently exhibited approximately 10–20% of the central chamber setpoint. This confirmed the presence of distinct ozone, low-ozone, and no-ozone exposure chambers. To avoid the likelihood of passive mite transport, extremely low air velocities (approximately 0.7–1.2 mm/s) across each chamber were maintained^17^. The structural design of the chamber system further reduced the likelihood of passive mite transport. The elevated interconnecting tunnels require mites to actively climb toward the tunnel entrance, traverse the connecting passage, and descend into the adjacent chamber. This created a physical behavioural barrier that minimized inadvertent displacement by airflow and ensured that inter-chamber migration reflected active movement rather than passive drift.

### Mobility assessments

The experiment was conducted to determine whether ozone exposure affects the mobility of *D. farinae* at varying concentrations (0.05 ppm, 0.5 ppm, and 1.0 ppm) over 72 h. Three connected acrylic chambers were used to create a continuous environment that allowed the mites to move freely between sections. Before the experiment, all chambers and lids were thoroughly cleaned using tissues and 75% ethanol. The ozone generator and ozone monitor were then switched on and allowed to stabilise for approximately 30 min before setting to the desired ozone concentration.

A T75 culture flask containing *D. farinae* was examined under a stereomicroscope to ensure that the mites were active and suitable for testing. Two card papers measuring 11 cm × 11 cm were prepared with double-sided adhesive tape attached along their edges, while another card paper measuring 15 cm × 15 cm was left without adhesive. The taped cards were placed in the low ozone chamber (left) and the no ozone chamber (right), while the untaped card was placed in the middle chamber, which served as the ozone exposure zone. The setup of the ozone treatment chamber used for studying dust mites’ mobility as shown in Supplementary Fig. S1.

A scoop (approximately 300.00 mg) of active mites was weighed using a Sartorius ME series electronic balance (Sartorius AG, Germany) with a precision of two decimal places and was later transferred onto a card in the middle ozone chamber, after which all chamber lids were securely closed to maintain airtight conditions. The ozone concentration was adjusted to the target level and maintained for 72 h, mites were allowed to move freely across the connected chambers, and those that reached the low-ozone or no-ozone chambers became trapped on the double-sided adhesive tape. After 72 h of exposure, the card papers from the low ozone and no ozone chambers were carefully removed and examined under a stereomicroscope. The number of mites trapped on each card was counted and recorded to evaluate the distribution of mites between the different ozone exposure chambers. Each ozone concentration test was performed in six replicates.

### Mortality assessments

The experiment was adapted from a previous study, with modifications^5^. A petri dish (10 cm in diameter) was placed into the mortality and protein-exposure chamber (Fig. 2). The petri dish was prepared by lining the base with filter paper and applying a thin layer of Vaseline along the inner wall and around the edge of the Whatman No. 1 filter paper to prevent mite escape. Approximately 100 active adult mites were gently transferred into each petri dish. Each ozone concentration (0.05 ppm, 0.5 ppm, and 1.0 ppm) was tested with three replicates per exposure duration (24, 48, and 72 h). Control groups were prepared in the same manner without turning on the ozone generator. After each experiment, the petri dishes were removed from the chamber, and the number of live and dead mites was recorded. Mites that remained motionless when gently probed with a wooden stick were considered dead.

### Protein extraction and western blotting

The setup used for the mortality assessment was employed with minor modifications. Approximately 1.00 g of active *D. farinae* was weighed using a Sartorius balance from T75 cultures after visually inspecting the culture to have contained > 95% eight-legged adult stages mites. The mites were then placed into a petri dish lined with Vaseline and were exposed to different ozone concentrations (0ppm, 0.05ppm, 0.5ppm, and 1ppm) for 24, 48, or 72 h. For protein analysis, six biological replicates were prepared per condition; three were pooled to generate one representative sample.

Following exposure, mites were homogenised in PBS containing protease inhibitor cocktail (Roche, Germany), and total protein was extracted for SDS-PAGE and Western blotting using a method adapted from our previous study^15^. BCA assay was performed using the Pierce™ BCA Protein Assay Kit (Thermo Fisher Scientific, USA). Equalised protein concentrations were resolved on 12.5% SDS–PAGE gels, transferred to PVDF membranes (Merck Millipore, USA) that were pre-activated in methanol for 8 min (18 V, 60 min), blocked with 5% BSA in TBST for 2 h in room temperature, and then probed with anti-Der f 1 antibody (1:1000 dilution) overnight at 4 °C (Sino Biological, China) to examine ozone-induced changes in the major *D. farinae* allergen. Additional probing was also performed using anti-*D. pteronyssinus* antibody, anti-*Tyrophagus putrescentiae* antibody, and anti-Blo t 5 antibody (InBio, US) to observe broader protein responses across different mite-related targets. The anti-*D. pteronyssinus* (anti-DP) and anti-*T. putrescentiae* (anti-TP) antibodies were obtained from our previous study^18^. The dilution ratio and condition remain the same as with probing with anti-Der f 1 antibody. Corresponding secondary antibodies (anti- mouse or anti-rabbit IgG, 1:10000, 1 h, RT) (Cusabio, China) were applied. The immune complexes formed were detected using ECL chemiluminescence (Nacalai Tesque, Japan) and imaged with a ChemiDoc System (Bio-Rad Laboratories, USA) under auto-exposure mode. Band intensities were quantified using Image Lab software v6.0 (Bio-Rad Laboratories, USA).

### *D. farinae* surface microbiota and bioinformatics analysis

*D. farinae* cultures were visually inspected to confirm > 95% eight-legged adult stages prior to weighing. Approximately 1.00 g of culture was used per replicate. The mites were then exposed for 72 h under four conditions: control (0 ppm), 0.05 ppm, 0.5 ppm, and 1.0 ppm, using the same ozone chamber as shown in Fig. 2. Three biological replicates were used per condition. After exposure, the mites and associated material from each dish were sieved through a cotton-gauze funnel into a 15 mL tube containing 8 mL PBS. The suspension was briefly vortexed and centrifuged at 1200 rpm for 3 min to detach surface microbiota. The resulting supernatant was aliquoted into 1.5 mL tubes and centrifuged at 15,000 rpm to pellet the bacterial fraction, which was retained for DNA extraction.

Genomic DNA was extracted using the Monarch^®^ Genomic DNA Purification Kit (New England Biolabs, USA) according to the manufacturer’s protocol. DNA purity and concentration were determined using a NanoDrop spectrophotometer (DeNovix Inc., USA).

Samples that met quality control standards were subjected to library construction according to the Illumina 16 S Metagenomic Sequencing Library Preparation protocol^19^. The V3–V4 hypervariable regions were amplified directly from genomic DNA using PCR, followed by ligation of adapters to the 5′ and 3′ ends of the amplicons. Adapter-ligated fragments were amplified using PCR primers targeting the V3–V4 regions with 25 cycles of Amplicon PCR using 2x KAPA HiFi HotStart ReadyMix (KAPA Biosystems, USA). A negative control, substituting nuclease-free water for the DNA template, was included throughout all steps. AMPure XP bead purifications (Beckman Coulter Genomics, USA) were performed after each PCR, followed by 8 cycles of Index PCR using the Nextera XT Index Kit (Illumina, USA). Final libraries were quantified using fluorometric dsDNA binding dyes, normalized to 4 nM in 10 mM Tris pH 8.5, pooled, and spiked with 5% PhiX Control v3 (Illumina, USA) before paired-end 300 bp sequencing on an Illumina MiSeq system (Illumina, USA) using MiSeq Reagent Kit v3 (600 cycles; Illumina, USA), targeting > 100,000 reads per sample. Raw images were processed by Real-Time Analysis (RTA) software (Illumina, USA) for base calling, and BCL files were converted to FASTQ for downstream analysis.

Sequence reads were imported into the CLC Genomics Workbench pipeline (Qiagen, Germany) for bioinformatics analysis. Following quality checking and trimming, operational taxonomic unit (OTU) clustering was performed for each region of all samples independently using the GreenGenes database, with clustering conducted at 97% sequence identity^20^. The flow chart illustrating the template workflow used for 16 S rRNA analysis in the CLC Genomics Workbench is shown in Supplementary Fig. S2.

### Statistical analyses

Statistical analyses were performed in Python (Google Colab) using the pandas, NumPy, SciPy, statsmodels, and matplotlib packages. A Preference Index (PI) was calculated to quantify mite distribution between the two chambers, following a two-choice behavioural metric adapted from Rharrabe et al. (2014) using a two-choice behavioural formula:

$$\:\mathrm{P}\mathrm{I}=\frac{({\mathrm{N}}_{{\mathrm{No\:O}}_{3}}-{\mathrm{N}}_{{\mathrm{Low\:O}}_{3}})}{({\mathrm{N}}_{{\mathrm{No\:O}}_{3}}+{\mathrm{N}}_{{\mathrm{Low\:O}}_{3}})}$$

where $\:{\mathrm{N}}_{{\mathrm{No\:O}}_{3}}$and $\:{\mathrm{N}}_{{\mathrm{Low\:O}}_{3}}$represent the number of mites in the no-ozone and low-ozone chambers, respectively^21^. The index ranges from − 1 to + 1, with positive values indicating preference for the no-ozone chamber and negative values indicating avoidance.

Differences in mite distribution between the paired chambers were analysed using paired t-tests. Mortality outcomes were compared using independent t-tests. Western blot band intensities were evaluated using one-way ANOVA with Tukey’s post-hoc test, while two-way ANOVA was applied to determine the effects of multiple factors. Statistical significance was set at *p* < 0.05.

## Results

### Mobility and mortality assessments

**Fig. 4:**
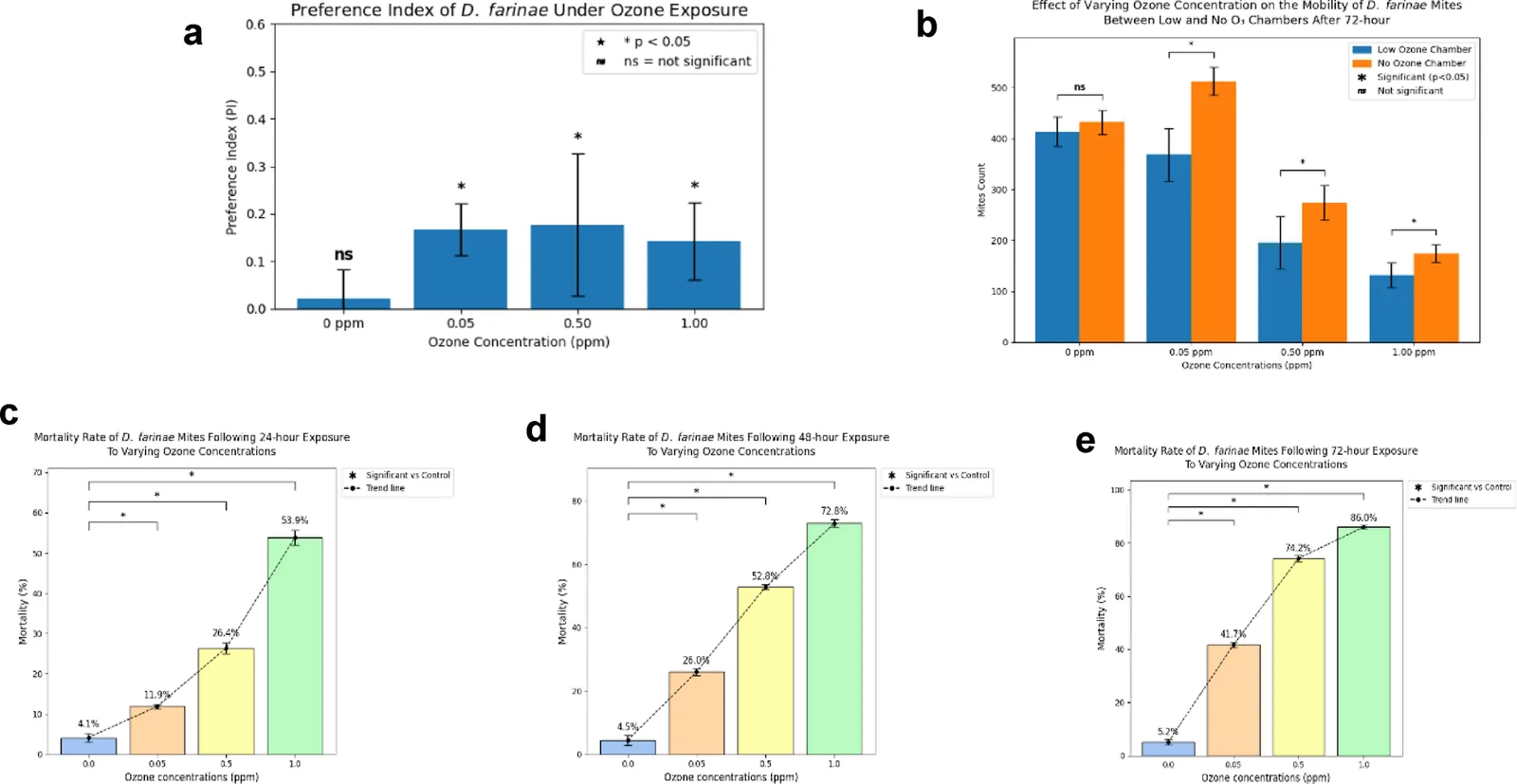
Effects of ozone exposure on *D. farinae* mobility and survival. (**a**) Preference Index (PI) of *D. farinae* under varying ozone concentrations (0, 0.05, 0.5, and 1.0 ppm). Bars represent mean PI values, and error bars indicate standard deviation. Asterisks denote statistically significant deviation from zero preference (*p* < 0.05), while “ns” indicates no significant difference. Positive PI values indicate a preference for the ozone-free chamber. A significant avoidance response is observed at all ozone-exposed conditions, whereas the 0 ppm control shows no significant preference, confirming unbiased baseline behaviour. (**b**) Mobility response of *D. farinae* following 72 h ozone exposure, showing mite distribution between ozone and ozone-free chambers. (**c**–**e**) Mortality rates of *D. farinae* after 24 h, 48 h, and 72 h of exposure to increasing ozone concentrations, demonstrating a time- and concentration-dependent rise in mortality.

Ozone exposure affected both the mobility and survival of *D. farinae*. In the mobility assay, mites consistently preferred the ozone-free compartment across all tested concentrations (0.05, 0.5, and 1.0 ppm). The Preference Index remained relatively consistent across ozone concentrations, ranging from 0.14 ± 0.08 to 0.18 ± 0.15 (mean ± SD) and the proportion of mites in the ozone-free chamber was significantly higher than in the ozone-exposed chamber at every concentration tested (*n* = 6; 0.05 ppm: *t* = 8.24, *p* = 0.0004; 0.5 ppm: *t* = 3.283, *p* = 0.0219; 1.0 ppm: *t* = 4.366, *p* = 0.0073) as shown in Fig. 4a. Total movement declined in a concentration-dependent manner, with the greatest reduction observed at 1.0 ppm. In contrast, the 0 ppm control showed no significant preference (PI = 0.02 ± 0.06; *p* = 0.423), confirming unbiased baseline movement. Overall activity declined with increasing ozone concentration, with the greatest reduction observed at 1.0 ppm. The effect of ozone concentration on mite mobility is shown in Fig. 4b.

Ozone exposure also induced an obvious concentration- and time-dependent increase in mortality. After 24 h, mean mortality reached 11.9 ± 0.5%, 26.4 ± 1.4%, and 53.9 ± 1.8% for 0.05, 0.5, and 1.0 ppm, respectively, compared with 4.1 ± 1.1% of the control (*n* = 3; all *p* < 0.001). Mortality further increased to 26.0 ± 1.0%, 52.8 ± 0.8%, and 72.8 ± 1.2% at 48 h, and reached 41.7 ± 1.0%, 74.2 ± 1.0%, and 86.0 ± 0.7% after 72 h (*n* = 3; two-way ANOVA, concentration effect *p* < 0.001; time effect *p* < 0.001). The results were summarised in Fig. 4c–e. All ozone-treated groups showed significantly higher mortality than the control at every time point (*p* < 0.001). These results confirm that increasing ozone concentration and prolonged exposure strongly reduce *D. farinae* survival.

### Western blot analysis

**Fig. 5:**
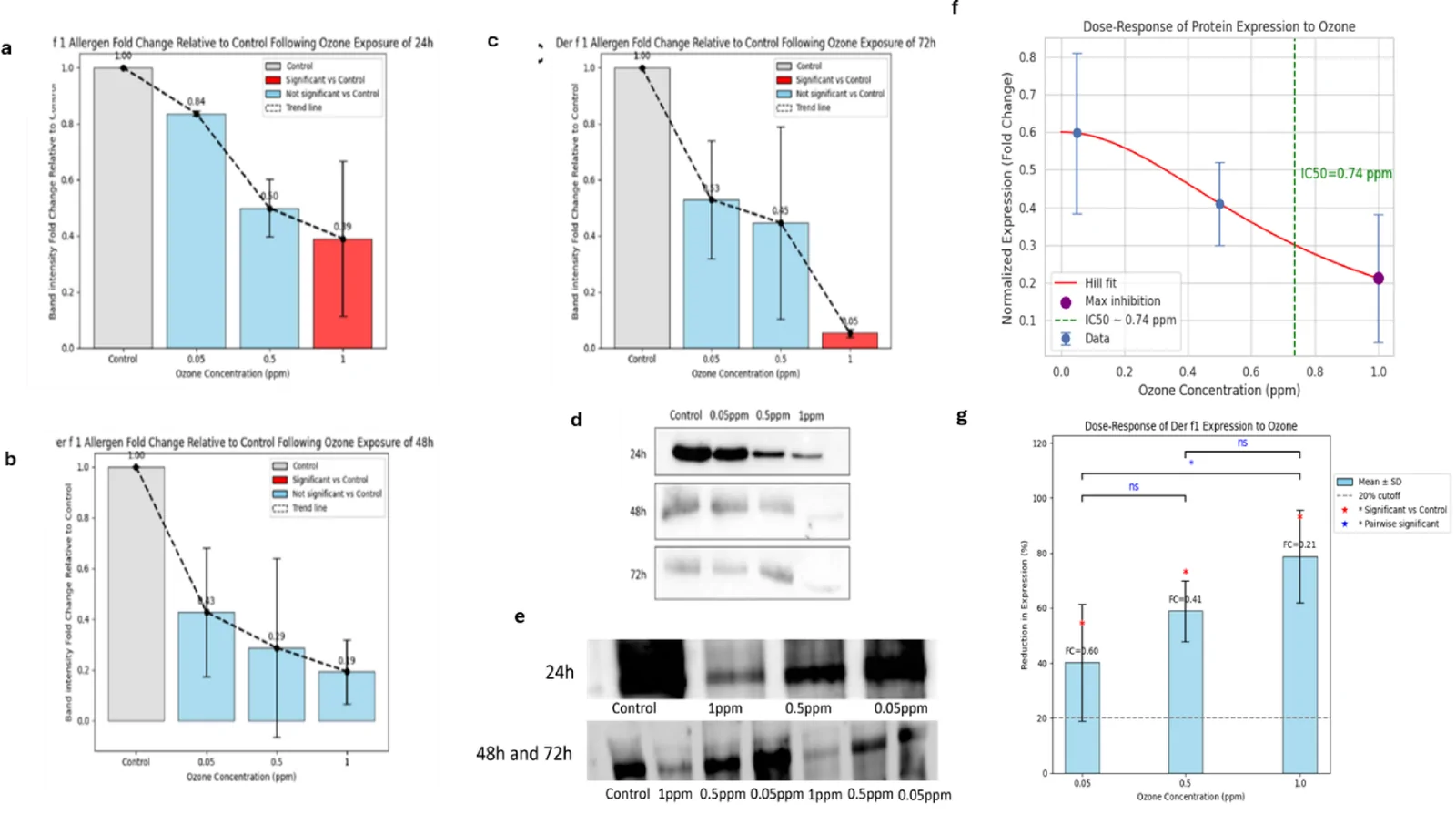
Ozone-induced changes in Der f 1 expression in *D. farinae*. (**a**–**c**) Fold-change in Der f 1 protein abundance following 24 h, 48 h, and 72 h of ozone exposure (0.05–1.0 ppm), relative to unexposed controls. Densitometric analysis (Bio-Rad Image Lab) revealed a concentration-dependent reduction in Der f 1 levels. The decrease was statistically significant at 24 h and 72 h (24 h: F(3,4) = 7.506, *p* = 0.0405; 72 h: F(3,4) = 7.473, *p* = 0.0408), while 48 h showed a downward trend that did not reach significance (F(3,4) = 5.123, *p* = 0.0742). Tukey’s HSD post-hoc test confirmed that the 1.0 ppm group differed significantly from the control at 24 h and 72 h (*p* < 0.05). Bars represent mean ± SD, and *p* < 0.05 was considered statistically significant. (**d**) Representative Western blot of Der f 1 (~ 25 kDa) at 24, 48, and 72 h under increasing ozone concentrations. (**e**) Immunoblot of ozone-exposed mites (24–72 h) probed with *D. pteronyssinus* hyperimmune sera. (**f**) Hill-curve model fitting Der f 1 expression to ozone concentration, illustrating the concentration–response relationship. (**g**) Percent Der f 1 expression across ozone concentrations, demonstrating the progressive reduction in allergen abundance.

Clear Der f 1 band in *D. farinae* extracts were observed when probed with the anti-Der f 1 antibody, with band intensity decreasing progressively as ozone concentration increased. Probing with anti-*D. pteronyssinus* (anti-DP) antibody also demonstrated ozone-associated alterations in protein signal. In contrast, no bands were detected when membranes were probed with anti-*T. putrescentiae* (anti-TP) or anti-Blo t 5 antibodies, confirming the absence or undetectable levels of these proteins in *D. farinae* extracts and the species-specific nature of the observed bands. The densitometry analysis and representative blot images are shown in Fig. 5a–e. Original blots are presented in Supplementary Figs. 3–6.

One-way ANOVA was performed to compare Der f 1 expression across ozone concentrations at each time point. At 24 h, ozone exposure produced a significant overall effect on Der f 1 levels (F = 7.506, *p* = 0.0405), and Tukey’s post-hoc test identified a significant reduction only at 1.0 ppm compared with the control (*p* = 0.0472). At 48 h, no significant differences were detected among treatment groups (F = 5.123, *p* = 0.0742), and all pairwise comparisons were non-significant. By 72 h, ANOVA again showed a significant effect of ozone concentration (F = 7.473, *p* = 0.0408), with post-hoc analysis confirming that only the 1.0 ppm group differed significantly from the control (*p* = 0.0309). Lower ozone concentrations (0.05 and 0.5 ppm) showed no significant changes at any time point.

Two-way ANOVA revealed that ozone concentration had a significant effect on the measured outcomes (*p* < 0.001), whereas exposure time did not produce a significant main effect (*p* > 0.05). No interaction between concentration and time was detected (*p* > 0.05), indicating that the impact of ozone was consistent across all time points. Post-hoc Tukey comparisons confirmed that only the highest concentration (1.0 ppm) differed significantly from the control, while 0.05 and 0.5 ppm groups showed no significant differences. These findings indicate that ozone concentration, rather than exposure duration, is the primary determinant of the observed protein response.

Since two-way ANOVA showed no significant effect of exposure time (24–72 h) or interaction between time and ozone, all time points were combined to calculate mean ± SD for each concentration. Figure 5f shows a Hill curve fit (red line) applied to quantify the dose-response relationship. The IC50 (green dashed line) indicates the ozone concentration causing ~ 50% reduction in protein expression. The maximum inhibition point is highlighted in purple. Figure 5g shows the percentage reduction in protein expression across ozone concentrations (0.05, 0.5, and 1 ppm) compared to the control. Error bars represent standard deviation. A horizontal dashed line indicates the 20% reduction cutoff. Red asterisks mark concentrations that are significantly different from control (Tukey HSD, *p* < 0.05).

### Ozone effects on *D. farinae* surface microbiota

**Fig. 6:**
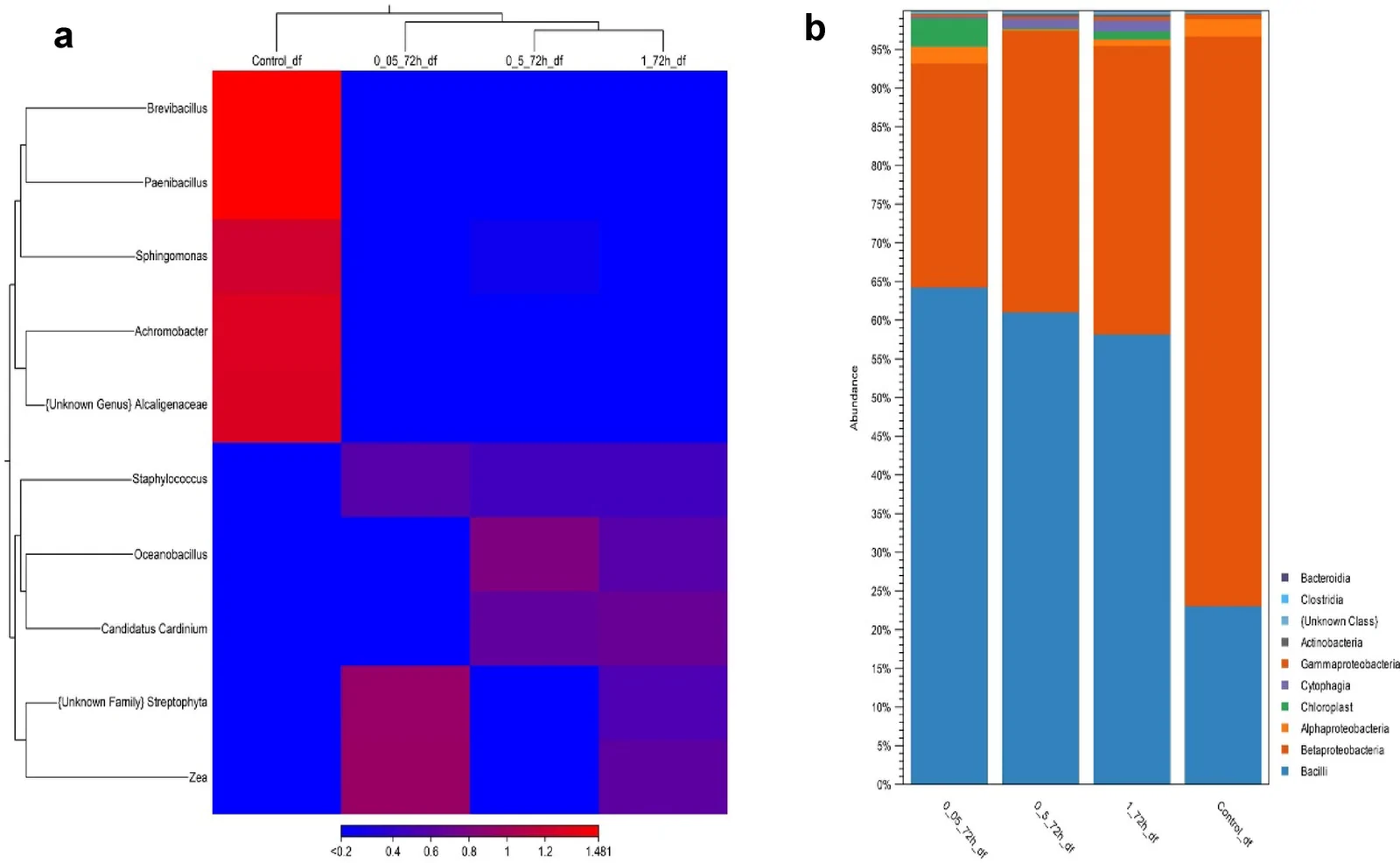
Ozone-induced shifts in the surface microbiota of *D. farinae*. (**a**) Hierarchical clustering heatmap of bacterial genera associated with *D. farinae* after 72 h of ozone exposure, illustrating distinct clustering patterns and compositional shifts in response to increasing ozone concentrations. (**b**) Relative class-level composition of the surface microbiota of *D. farinae* after 72 h of ozone exposure, showing changes in bacterial community structure following ozone treatment.

16 S rRNA gene sequencing revealed clear compositional shifts in the bacterial community associated with *D. farinae* following 72 h of ozone exposure. In control samples, the most abundant genera were *Brevibacillus*, *Paenibacillus*, *Sphingomonas*, *Achromobacter*, and an unclassified *Alcaligenaceae* genus (Fig. 6a). Their relative abundance declined progressively at 0.05, 0.5, and 1 ppm ozone, with the strongest reduction observed at 1 ppm. Low-abundance genera such as *Staphylococcus*, *Oceanobacillus*, and *Candidatus Cardinium* were detected mainly in ozone-treated samples. At the class level, the control group’s microbiota consisted predominantly of *Bacilli* and *Alphaproteobacteria*, with minor contributions from *Actinobacteria* and *Clostridia* (Fig. 6b). After ozone exposure, the relative proportion of *Bacilli* decreased with increasing ozone concentration, whereas *Alphaproteobacteri*a and *Betaproteobacteria* became more represented. These community-level changes were consistent across all ozone-treated conditions and distinct from the control profile, indicating a clear ozone-associated alteration in the surface microbiota composition of *D. farinae* mites.

## Discussion

Ozone exposure influences *D.farinae* behaviour, survival, allergen production, and the composition of surface-associated microbiota. Our findings provide compelling evidence that ozone can induce both sublethal and lethal biological consequences in HDMs and may, under controlled conditions, serve as a potential strategy against mites and their associated allergenic burden. The ozone concentrations were chosen to represent a realistic-to-elevated exposure range. The lowest concentration (0.05 ppm) corresponds to the maximum level permitted in indoor environments by regulatory bodies such as the US Environmental Protection Agency, and reflects the typical output of consumer ionizers and ozone-generating purifiers^22^. Higher ozone concentrations (0.5 and 1.0 ppm) were included because values within this range are commonly used in controlled laboratory investigations examining ozone’s biological effects on mites and other small arthropods, providing a benchmark for experimental comparisons across previous oxidative-stress and fumigation studies^23–25^. The exposure durations of 24, 48, and 72 h were selected to capture acute and early sub-acute biological responses, aligning with established definitions of short-term ozone exposure and reflecting time frames previously used to demonstrate cumulative oxidative effects on arthropod physiology^5,22,26^.

Behavioural assays revealed a consistent avoidance response across all tested ozone concentrations. Mites preferentially migrated toward the ozone-free compartment, reflected by a stable Preference Index, indicating that *D. farinae* can detect and actively avoid oxidative environments. However, mobility decreased at higher ozone levels, suggesting that early sublethal effects, such as oxidative stress, neuromuscular impairment, or reduced energy metabolism, may limit sustained movement, as similarly reported in other oxidant-exposed arthropods^27–29^. Mortality patterns further supported a concentration-dependent effect, with extended exposure to 1.0 ppm leading to over 85% mite death by 72 h. Ozone-induced oxidative damage to lipids, proteins, and cuticular structures likely contributed to the progressive lethality^25,26^. Compared with previous ioniser-based studies, the present experiment showed a faster and more pronounced lethal effect, most likely due to the controlled chamber design, real-time ozone monitoring, and homogenous and direct exposure, which minimised fluctuation and ensured stable ozone delivery^5^.

In addition to behavioural and survival impacts, ozone exposure significantly suppressed the expression of the major allergen Der f 1 in a concentration-dependent manner. Der f 1 was chosen as the primary target due to its high prevalence in Southeast Asia, being more commonly detected than Der p 1 in Malaysia, Thailand, and Vietnam^30^. As a cysteine protease that is abundant in mite faeces and highly stable in indoor environments, Der f 1 can disrupt epithelial barriers and strongly drives allergic sensitisation, making it the most clinically relevant marker for assessing ozone-mediated allergen reduction^31^.

Although one-way ANOVA at individual time points initially suggested that only 1.0 ppm had a significant effect, two-way ANOVA confirmed that ozone concentration, rather than exposure duration, was the primary driver of allergen suppression. Notably, even low-level ozone (0.05 ppm), corresponding to indoor safety recommendations, showed reduced Der f 1 expression when data were pooled across time points. This outcome is biologically relevant because Der f 1 is a cysteine protease with a redox-sensitive active site^32,33^. Oxidation of its thiol groups may disrupt tertiary structure, inhibit proteolytic function, and interfere with its ability to cleave epithelial junction proteins such as ZO-1 and occludin, which are the key steps in allergen sensitisation^34^. Thus, ozone-mediated suppression of Der f 1 may theoretically reduce allergenic potential at the source, aligning with its concentration-dependent acaricidal effects. Additional protein band changes observed with anti–*D. pteronyssinus* sera suggest broader ozone-induced proteomic alterations, likely due to the close phylogenetic relationship between *Dermatophagoides* species^35^.

Furthermore, 16 S rRNA sequencing indicated that ozone exposure reshaped the surface microbiota of *D. farinae*, even at 0.05 ppm. Mite-associated bacteria can contribute lipopolysaccharides and other microbe-associated molecular patterns (MAMPs) that potentiate Th2-type immune responses^36–38^. As such, ozone-driven microbiome shifts may modify allergen–microbe interactions and influence downstream host inflammation^39,40^. This finding is significant in the context of household air-purification devices, which can generate low-level ozone. Depending on which taxa are suppressed or enriched, ozone exposure may reduce, maintain, or potentiate allergenicity.

Several limitations must be acknowledged. Although ozone exposure induced a clear concentration- and time-dependent increase in *D. farinae* mortality, this effect likely confounds the interpretation of downstream outcomes such as Der f 1 levels and microbiome composition. The substantial reduction in mite viability, particularly at higher ozone concentrations, may have contributed indirectly to the observed decreases in allergen abundance and alterations in bacterial communities through reduced mite biomass and post-mortem changes. Since the precise mechanism by which ozone exerts its acaricidal effects on *D. farinae* remains unknown, this current study could not fully disentangle direct ozone-mediated biochemical effects from indirect consequences of mite mortality. Moreover, biological replicates for Western blotting and microbiome analysis were pooled, preventing statistical assessment of inter-sample variation. Laboratory-maintained mite colonies may not fully represent the heterogeneous microbiota or stress responses of environmental mite populations. In addition, the study quantified protein expression but not protease activity; thus, the inhibitory effects observed likely underestimate the full functional impact of ozone, as inactivation can occur without reduced protein abundance. While our results demonstrate that mites actively avoid ozone and that ozone exposure leads to protein carbonylation and reduced allergen levels, it is still unclear how ozone is initially detected by the mites. One explanation is that ozone acts through the chemosensory system^41^. Ozone, as a strong oxidant, may directly interact with these chemoreceptors or indirectly alter the chemical microenvironment, triggering an aversive signal. However, direct evidence for ozone binding to mite chemoreceptors is currently lacking.

Despite these limitations, the present study provides integrated evidence that ozone can impair mite mobility, induce mortality, and is associated with reduced allergen levels and altered microbial communities. These combined effects highlight ozone’s potential as a non-chemical environmental intervention in reducing mite-related allergen burden, particularly in enclosed indoor settings. However, because ozone is itself a respiratory irritant, any practical application must be carefully balanced against human safety guidelines^42^. Future research should employ mite activity assays, proteomics, metagenomics, and immunological models to determine whether ozone-driven biochemical and microbiome changes translate into reduced allergenicity in vivo^4^.

## Conclusion

In conclusion, our study demonstrated that ozone exposure exerts many effects on *D. farinae*, particularly influencing their behaviour, survival, allergen production, and surface-associated microbiota. Mite mobility was consistently reduced in ozone-treated conditions, with mites strongly avoiding ozone-containing chambers, while mortality increased in a concentration- and time-dependent manner, indicating that ozone impairs both activity and survival. At the molecular level, ozone exposure caused a clear, concentration-dependent suppression of the major allergen Der f 1, with even low-level exposure (0.05 ppm) producing measurable reductions, suggesting potential mitigation of allergenic potential. Furthermore, 16 S rRNA gene analysis revealed that ozone modulated the surface microbiota of the mites, altering bacterial community composition even at indoor-safe ozone levels. Taken together, these findings provide comprehensive evidence that ozone impacts *D. farinae* across behavioural, molecular, and microbiological dimensions, highlighting its potential as a controlled, non-chemical strategy in reducing HDMs allergen burden and associated health risks in indoor environments.

## Supplementary Information

Below is the link to the electronic supplementary material.

Supplementary Material 1

## Data Availability

The raw sequencing data generated in this study have been deposited in the NCBI Sequence Read Archive (SRA) under BioProject accession number PRJNA1354801 ([https://www.ncbi.nlm.nih.gov/bioproject/1354801](https:/www.ncbi.nlm.nih.gov/bioproject/1354801)). The corresponding BioSample accession numbers are SAMN52955989, SAMN52955990, SAMN52955991, and SAMN52955992. All other data supporting the findings of this study are available from the corresponding author upon reasonable request.
