# Supplementary material for: Effects of ozone exposure on house dust mites
Source: Sci Rep. 2026 Jun 22;16:28520. doi: 10.1038/s41598-026-59397-8 (PMC13572548; doi:10.1038/s41598-026-59397-8)
Supplement: Supplementary file 1 [file 41598_2026_59397_MOESM1_ESM.docx]

**Supplementary Figure S1.** Set-up of ozone treatment chamber for mobility test.

**Supplementary Figure S2.** QIAGEN CLC Microbial Genomics Module.

**Supplementary Figure S3.** Full-length, uncropped Western blot for Der f 1 (~25 kDa) at 24 hours. This figure shows the full, original Western blot corresponding to the cropped representative image presented in the main text (Figure 7d). All lanes, including the molecular weight ladder (1st BASE, Malaysia), are displayed in their entirety. Samples include fish-flake vehicle controls to account for potential background signals introduced by the culture substrate, along with *D. farinae* mites exposed to increasing ozone concentrations at 24 hours. No image- specific alterations, splicing, or selective adjustments were made; only uniform brightness/contrast adjustments were applied to the entire image. This full-length blot is provided to comply with the journal’s digital image and integrity policy.

**Supplementary Figure S4.** Full-length, uncropped Western blot for Der f 1 (~25 kDa) at 48 hours and 72 hours. This figure shows the full, original Western blot corresponding to the cropped representative image presented in the main text (Figure 7d). All lanes, including the molecular weight ladder (Simply Biologics, Malaysia), are displayed in their entirety. *D. farinae* mites were exposed to increasing ozone concentrations at 48 hours and 72 hours. No image-specific alterations, splicing, or selective adjustments were made; only uniform brightness/contrast adjustments were applied to the entire image. This full-length blot is provided to comply with the journal’s digital image and integrity policy.

**Supplementary Figure S5.** Full-length, uncropped Western blot of ozone-exposed mites (24 h) probed with *D. pteronyssinus* hyperimmune sera. This figure shows the full, original Western blot corresponding to the cropped representative image presented in the main text (Figure 7e). All lanes, including the molecular weight ladder (1st BASE, Malaysia), are shown in their entirety. Samples include fish-flake vehicle controls to account for potential background signals from the culture substrate, along with *D. farinae* mites exposed to increasing ozone concentrations for 24 hours. No image-specific alterations, splicing, or selective adjustments were made; only uniform brightness and contrast adjustments were applied to the entire image. This full-length blot is provided to comply with the journal’s digital image integrity policy.

**Supplementary Figure S6.** Full-length, uncropped Western blot of ozone-exposed mites (48 hours and 72 hours) probed with *D. pteronyssinus* hyperimmune sera. This figure shows the full, original Western blot corresponding to the cropped representative image presented in the main text (Figure 7e). All lanes, including the molecular weight ladder (1st BASE, Malaysia), are shown in their entirety*. D. farinae* mites were exposed to increasing ozone concentrations for 48 hours and 72 hours. No image-specific alterations, splicing, or selective adjustments were made; only uniform brightness and contrast adjustments were applied to the entire image. This full-length blot is provided to comply with the journal’s digital image integrity policy.
